# A Conflict of Testimony

**Published:** 1919-10-04

**Authors:** 


### THE EDITOR'S LETTER-BOX.

**[Correspondence on all subjects is invited, but we cannot in any way be responsible for the opinions expressed by our correspondents, who must give their name and address as a guarantee of good faith, but not necessarily for publication. Correspondents are reminded that brevity of style and conciseness of statement greatly facilitate early insertion.]**

### QUEEN CHARLOTTE'S HOSPITAL AND ITS NURSING STAFF.

### A Conflict of Testimony.

*To the Editor of* The Hospital.

Sir,—I wish to thank you for your letter of generous support of the pupil midwives at the above hospital, which came out in The Hospital of September 20. I would like to say that all the facts published, both in your letter and the nurses', are absolutely correct. There is only one very important fact not mentioned by either of you, and that is the utter incivility, and, to put it mildly, unkindness of practically every member of the staff to the pupils, from the matron down to the wardmaids. This accounts for most of the resignations on the part of pupils, and a demand for their money back. Unless the pupil leaves within the first month of her training she never sees her money again, however good her case may be. Thanking you again for your support,

Yours truly, P. C.

Queen Charlotte's Hospital,
Marylebone Road, N.W.
*September* 23, 1919.

